# SELDI-TOF-MS Serum Profiling Reveals Predictors of Cardiac MRI Changes in Marathon Runners

**DOI:** 10.1155/2012/679301

**Published:** 2012-09-03

**Authors:** George D. Wilson, Timothy J. Geddes, Barbara L. Pruetz, Bryan J. Thibodeau, Amy Murawka, James M. Colar, Peter A. McCullough, Justin E. Trivax

**Affiliations:** ^1^Beaumont BioBank, William Beaumont Hospital, Royal Oak, MI 48073, USA; ^2^Department of Radiation Oncology, William Beaumont Hospital, Royal Oak, MI 48073, USA; ^3^Department of Cardiovascular Medicine, William Beaumont Hospital, Royal Oak, MI 48073, USA

## Abstract

*Purpose*. To utilize proteomics to discover proteins associated with significant cardiac magnetic resonance imaging (MRI) changes in marathon runners. *Methods*. Serum from 25 runners was analyzed by surface enhanced laser desorption ionization time-of-flight mass spectrometry (SELDI-TOF-MS). Proteomic profiles were compared in serum samples obtained prior to the race, at the finish line and within 7 hours after race to identify dynamic proteins correlated with cardiac MRI changes. *Results*. 693 protein/peptide clusters were identified using two ProteinChip surface chemistries and, of these, 116 were significantly different between the three time points. We identified 7 different patterns of protein expression change within the runners and 5 prerace protein peaks, 16 finish-line protein levels, and 15 postrace proteins which were correlated with significant postrace cardiac MRI changes. *Conclusions*. This study has identified baseline levels of proteins which may be predictive of risk of significant cardiac damage following a marathon race. Preliminary identification of the significant proteins suggested the involvement of cytokines and other proteins involved in stress and inflammatory response.

## 1. Introduction

A central paradox of physical activity is the association of moderate exercise with decreased cardiovascular morbidity and mortality [1, 2], whereas vigorous physical exertion increases the short-term risk of sudden cardiac death [3, 4]. Due to the rising participation in endurance sports, there is much current interest in postexercise changes in cardiac function [5]. Ever since the first marathon runner, Phidippides, collapsed and died at the finish of his famous run in ancient Greece, death due to heart-related stress has been an occasional occurrence in marathon events. Sudden unexpected death during marathons and other high impact activities is usually due to underlying and often unsuspected heart disease [6–8] and such catastrophes often attract substantial public attention [9]. Identification of marathon runners at risk is difficult, and the need for medical examinations remains controversial [2, 10, 11]. The risk of sudden cardiac death associated with marathon running has been suggested to be too low to recommend routine screening for coronary artery disease [12, 13]. An alternative approach to cardiac screening is to identify biomarkers which may predispose runners to increased risk of cardiac events.

Many physical and biochemical changes have been described during endurance sports including transient changes in systolic and more persistent diastolic dysfunction (as determined by MRI) of both the left (LV) and right ventricle (RV) [14–16] and consistent increases in cardiac biomarkers troponin [17–19] and B-type natriuretic peptide (BNP) [18, 20, 21]. However, none of the pre-race levels of these biomarkers can predict the post-race changes in cardiac function. Surface enhanced laser desorption ionization time-of-flight mass spectrometry (SELDI-TOF-MS) is a widely used tool to identify specific markers for diseases and certain physiological conditions. SELDI-TOF-MS profiling has been successfully applied to various cardiovascular disease studies [22–24]. In this study, SELDI-TOF-MS was used to discover differentially expressed proteins associated with increased risk of cardiac dysfunction prior to, during, and after a marathon event. This discovery study was an adjunct to a recently published investigation into traditionally accepted biochemical indices and physiological indices of cardiac stress, such as acute and chronic right ventricular dysfunction, heart chamber morphology and function, edema, tissue perfusion, dynamic myocardial contraction, and cardiac blood flow as determined by cardiovascular magnetic resonance [25].

## 2. Methods

### 2.1. Subject and Sample Description

This study was reviewed and approved (HIC #08-254) by Beaumont Hospital's Human Investigation Committee. From 425 volunteers, twenty-five (12 male and 13 female) were randomly selected and consented for the study. The details of the physiological, biochemical and imaging studies have been previously described [25]. Briefly, each volunteer provided a medical history and a description of their training. Baseline screening included cardiopulmonary exercise testing, blood biomarker analysis, cardiovascular MRI, and 24-hour ambulatory electrocardiography 4 weeks before (pre-race) and within 7 hours after race. The actual mean time for the post-race MRI was 4.03 ± 1.62 hours (range 0.83–7.02 hrs). Blood samples were drawn at the pre- and post-race MRI procedures but also at the finish line of the race. The time from blood draw through processing and freezing ranged from 40 minutes to 135 minutes with an average of draw to freeze time for all three samplings of 66.8 minutes. This included a 30 min ambient incubation to allow for clotting. Blood drawn at the finish line was processed at the site of the race and samples were frozen on dry ice until returned to the lab and stored at −80°C. Plasma and serum were separated but only serum was used in the SELDI analysis described in this study. The samples were analyzed by SELDI within three months of collection.

### 2.2. SELDI-TOF-MS Peak Generation

Discovery proteomics was performed using a ProteinChip SELDI-TOF-MS, Enterprise Edition (Bio-Rad Laboratories, Hercules, CA, USA). Two protein chip types with different binding chemistries were selected for the analysis. These were CM10 (weak cation exchange) and IMAC-30 (immobilized metal affinity chromatography).

Runner identification and sampling times were de-identified and assigned a barcode using BIGR (Healthcare IT, Rockville, MD, USA). For SELDI array spotting, samples were applied using the ProteinChip software randomizer function and were blinded at this point. Once spectra were generated, sampling time was assigned in order to perform comparative analysis.

Briefly, serum samples were thawed on ice then centrifuged to clear, after which 20 *μ*L of supernatant was denatured by the addition of 30 *μ*L of U9 buffer (9 M urea, 2% CHAPS, 50 mM Tris-HCI, 1% DTT, PH 9.0) (Bio-Rad). The samples were incubated on ice for 30 min and subsequently 200 *μ*L of 25 mM Tris pH 7.4 was added. Next, 2 *μ*L of sample was spotted in duplicate, after randomization, onto an array with 4 *μ*L of pre-spotted low stringency binding buffer (0.1 M sodium acetate, PH 4.0). Proteins were allowed to bind in a humid chamber for 30 minutes after which liquid was removed and the spots allowed to dry. Next, 1 *μ*L of saturated sinapinic acid (SPA) in 50% (V/V) acetonitrile and 0.5% trifluoroacetic acid was applied as an energy absorbing matrix and allowed to crystallize by solvent evaporation for 10 minutes at room temperature. After another addition/drying of 1 *μ*L SPA, the arrays were ready for SELDI data acquisition. Arrays of each chip type were bombarded under two laser intensity conditions (low energy 1600 nJ and high energy 3500 nJ) to more closely analyze lower and higher mass ranges. Each spot was divided into 50 partitions (4 pixels per partition), and each selected pixel was bombarded 12 times (the first two being “warming shots” at the selected energy + 10%). For the 1600 nJ condition, energy source was set at 25 kV, positive ions, mass range 0–20,000 Da, focus mass = 5,000 Da, matrix attenuation = 1000, sampling rate = 800 MHz. Under 3500 nJ bombardment, settings were adjusted to mass range = 0–200,000 Da, focus mass 16,000, and matrix attenuation = 5,000. This resulted in the generation of 640 individual spectra. Multiple aliquots of a pooled reference sample consisting of a mixture of all the samples were also spotted onto the ProteinChips for quality control, standardization, and normalization purposes.

### 2.3. Spectra Analysis

Analysis was performed using ProteinChip Data Manager 3.5 (Bio-Rad Laboratories, Inc., Hercules, CA, USA). Once accumulated, spectra were subject to peak detection, and qualified mass peaks were then defined by first creating and applying mass calibration equations using Bio-Rad's All-in-One peptide standards (low energy condition) or All-in-One protein standards (high energy condition). Peak intensity calculations were subsequently adjusted after baseline subtraction (fitting width set at 10 times the expected peak width using the smoothing function set at 25 points for the low mass range and 10 points for the high mass range). Next, noise was reduced by adjusting the average filter to 0.2 times the expected peak width. Peak intensities within a condition were subsequently normalized to total ion current to compensate for spectrum-to-spectrum variations, and outlying spectra were removed from the analysis. Spectra were then aligned to a reference-pool sample with a normalization factor of 1. Peak clustering was then carried out to group peaks of similar mass across multiple spectra using automatic first-pass peak detection settings of signal to noise > 2.0. Peak clusters were further defined using second-pass peak detection (signal-to-noise ratio >2, valley depth = 2.0) with minimum peak threshold set at 15% and a mass window approximately 0.1% of the peak mass. Estimated peaks were added to ensure that every spectrum is represented in the cluster. After peak clustering, peaks were subjected to statistical analysis with *P* values calculated across each group. Those clusters showing significant differences (*P* value ≤ 0.05) in the univariate analysis were compared against protein databases to identify potential candidate biomarkers. To afford a wide search range of proteins to choose from, a broad selection of pI range was explored along with a m/z value within ±0.1 decimal place of the observed value to account for posttranslational modifications such as phosphorylation and methylation. From this starting point, physical characteristics were selected by their potential to play a role in cardiac function, to be involved in known cardiopathologies and if the candidate was a secretory protein likely to be present in serum. By design, this investigation was to yield a broad range of m/z candidates for further investigation.

### 2.4. Statistical Analysis

Identification of proteins/peptides that demonstrated significant changes across the three specimen collection times was carried out using ANOVA using a *P* value of ≤0.05 to indicate significance. To establish significant changes between runners who did or did not experience a significant cardiac MRI change in right ventricular efflux volume (RV EF) after race, a 2-way ANOVA model using the method of moments was used.

## 3. Results

### 3.1. Peak Detection

A total of 693 protein/peptide clustering peaks >1 kDa were detected using the four different conditions (two different chip types at two different laser intensities). The number of protein/peptide peak clusters was 124 on CM10 (low energy), 334 on CM10 (high energy), 133 on IMAC30 (low energy), and 92 on IMAC (high energy) (See Figure, SDC 1, representative spectra from the two SELDI protein chip binding surfaces were utilized in this study which were bombarded under two laser intensity conditions (1) CM10 1600 nJ, (2) CM10 3500 nJ, (3) IMAC30 1600 nJ and (4) IMAC30 3500 nJ).

The peak intensities of 116 of the 693 protein/peptide clusters were significantly different at the three different time points studied as compared to an “all sample” pooled control. The CM10 surface chemistry identified many more significantly altered proteins than the IMAC-30 chip. This chip identified 22 proteins that were significantly altered (*P* ≤ 0.05) in the pre-, immediately post-race, and 2nd MRI samples whilst 73 were identified on the high laser power chip. In contrast, only 12 and 9 proteins were identified on the IMAC-30 chip. Twelve of these proteins were identified across different combinations of chip and laser power (see Tables, SDC 1–4 for the m/z ratios of proteins that were significantly different within each protein surface and laser power condition).

### 3.2. Patterns of Protein Change

**Figure 1:**
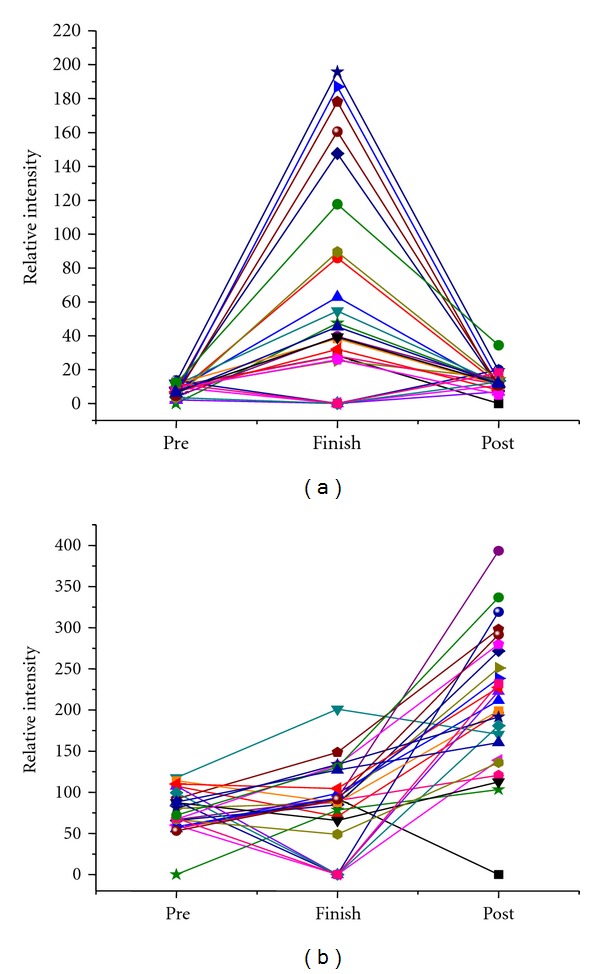
Representative examples of protein changes. (a) shows an example of pattern 1 in a protein with m/z ratio of 10840 interrogated with 1600 nJ on a CM10 chip for all 25 runners. (b) shows pattern 4 changes in a protein of m/z 11699 interrogated with 3500 nJ on a CM10 chip for all 25 runners.

We identified 7 different patterns of protein expression change within the runners (Table 1). 38% of significantly changed proteins showed an inverted “V” pattern that is, low levels before race, high levels at the finish line and reversion to low levels by the 2nd MRI (pattern 1). The opposite pattern, that is, “V” was seen in 20% of proteins (pattern 2). A progressive increase in protein levels was seen in 10% of the proteins (pattern 3), and 25% of proteins showed no change between pre-race and finish line but rose significantly at the second MRI (pattern 4). 5% showed the opposite pattern with similar levels pre-race and finish line but a drop at the second MRI (pattern 5).  Figure 1 shows an example of pattern 1 and pattern 4 across the 25 runners. In both cases, even though the overall trend followed the described pattern, there was individual variation between the runners and some deviated from the overall pattern; this was true for many of the significantly changed peaks.

### 3.3. Proteins Associated with Significant Cardiac MRI Changes

**Figure 2:**
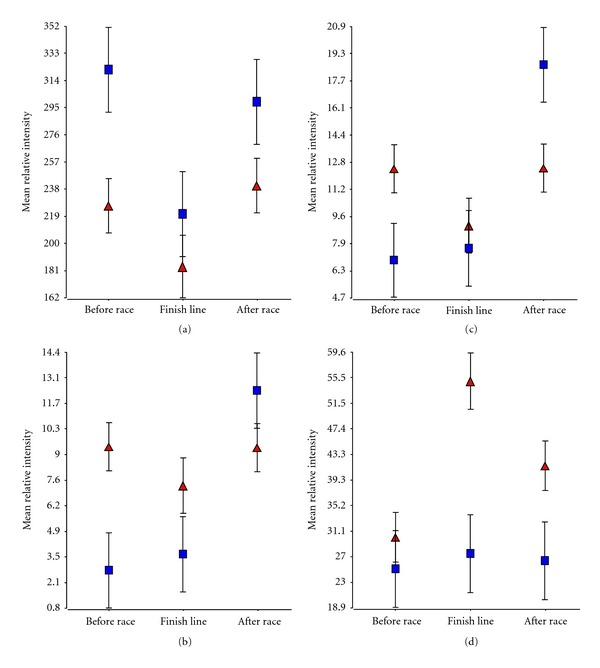
Examples proteins with significant expression changes associated with a significant change in post-race RV EF. (a) shows a relatively abundant protein of m/z 8922.3 in which a low pre-race level was associated with the runners at risk of RV EF reductions whilst (b) shows a lower abundance protein of m/z ratio 17897.5 in which a higher pre-race level predicted risk of RV EF reductions. (c) and (d) show two proteins that exhibited significant alterations at two of the three observation times. (c) shows a less abundant protein (m/z = 17772.3) in which a high pre-race level was associated with at risk runners whilst a lower post-race level was associated with runners who experienced significant RV EF changes. (d) represents a moderately abundant protein of m/z 10418.6 in which significant elevations at the finish line and at the post-race MRI were associated with RV EF reductions. ▲= at risk of RV EF reductions, ■ = no significant changes.

Based on the previous data [25], 17 of the 25 runners were demonstrated to have significant changes in their right ventricular efflux fraction (RV EF) that was considered to be the most important clinical change. When runners were dichotomized into these two groups, we identified 5 proteins whose pre-race level significantly predicted post-race RV EF changes, 16 proteins whose finish-line levels were associated with RV EF changes, and 15 proteins whose levels at the second MRI were associated with RV EF reductions.  Figure 2(a) shows a relatively abundant protein of mass charge ratio (m/z) 8922.3 in which a low pre-race level was associated with the runners at risk of RV EF reductions (*P* = 0.009) whilst Figure 2(b) shows a lower abundance protein of m/z ratio 17897.5 in which a higher pre-race level predicted risk of RV EF reductions (*P* = 0.0074). Four of the 5 pre-race proteins associated with significant RV EF changes were elevated in the “at risk” runners, and 4 of 5 exhibited pattern 2 of expression changes (Table 1). Only two proteins showed significant changes in two of the three time observations. In Figure 2(c), high pre-race levels (*P* = 0.042) of a less abundant protein of m/z = 17772.3 was associated with at risk runners whilst a lower post-race level (*P* = 0.023) was associated with runners who experienced significant RV EF changes. In Figure 2(d), a moderately abundant protein of m/z 10418.6 showed significant elevations at the finish line (*P* = 0.001) and at the post-race MRI (*P* = 0.043) and was associated with RV EF reductions.

At the finish line, 9 of the 16 proteins significantly altered peaks were elevated in the “at risk” runners, and 14 of these proteins exhibited pattern 1 responses overall. 10 of the protein peaks were significantly altered with *P* values less than 0.01 (Table 2).

At the post-race MRI time point, 10 of the 15 significantly altered protein peaks were raised in the runners who were found to have RV EF reductions. In contrast, to the other two time points, proteins changes at this time point were predominantly pattern 4 (7/15) and pattern 2 (6/15). The changes tended to be more subtle with only three protein peaks being significant at less than the 0.01 level (Table 2).

### 3.4. Preliminary Identification of Protein Candidates

Of the significantly different clustered protein peaks from specified time points in the study which correlated runner's cardiac stress based on MRI findings, candidates were chosen after comparing m/z ratios against the UniProtKB/Swiss-Protein database using the TagIdent tool available from the Swiss Institute of Bioinformatics' EXPASY proteomics server (Table 2). Although tentative, several proteins potentially associated with cardiac and systemic response to stress were consistent with the m/z ratios and chromatographic characteristics of the significantly altered SELDI-TOF peaks.

## 4. Discussion

The SELDI-TOF-MS technology implemented in the ProteinChip system is designed to perform MS analysis of protein mixtures retained on chromatographic array surfaces. Easily obtainable clinical biofluid samples, such as blood, urine or saliva can be directly applied to the ProteinChip array surface [26]. An advantage of SELDI-TOF-MS is its relatively high tolerance for salts and other impurities, also the complexity of the samples is reduced as proteins and any contaminants that do not bind to the spot surface are removed. In this study, SELDI-TOF-MS analysis revealed biomarkers with m/z ratios which had dynamic and significantly changed profiles before, at the finish line and up to 7 hours after running a marathon.

Although the identification of the significantly altered proteins in this study has yet to be confirmed by further mass MS analysis and validation by ELISA, bead array or western blotting, there is compelling evidence from the exercise literature that cytokines and other proteins involved in the inflammatory response are key players in the response to strenuous exercise [27, 28]. However, to our knowledge, this is the first study that has potentially implicated pre-race levels of cytokines as predictors of significant cardiac changes following a marathon race.

Specific changes, both after strenuous exercise and in infectious disease states [29], include the acute phase response, leukocyte mobilization and activation, release of inflammatory mediators (cytokines), tissue damage and cell infiltration, the production of free radicals and activation of the complement, coagulation, and fibrinolytic pathways. Brenner and colleagues showed that prolonged exercise induces a significant increase in IL-6 and tumor necrosis factor *α* plasma levels, the mobilization of cytotoxic cell populations and increased natural killer cell cytotoxic activity, all suggesting that prolonged exercise was effective in activating several components of the inflammatory response [29]. Cytokines are potent intercellular signaling molecules that regulate inflammation and immune responses by acting locally at extremely low concentrations in a paracrine or autocrine manner. A systemic inflammatory response syndrome (SIRS) can be elicited by a variety of serious insults such as severe trauma, burns, hemorrhagic shock, sepsis, and ischemia/reperfusion injuries and exhaustive exercise [30]. Nieman and coworkers have shown that four key cytokines, IL-6, IL-1ra, IL-10, and IL-8, are increased for distances up to the 26.2 mile marathon race [27]. However, these are among many pro- and anti-inflammatory cytokines and factors that are secreted systemically after exhaustive exercise or eccentric exercise [31–35]. Interestingly, three of the 5 proteins whose pre-race levels among the 25 runners were predictive of post-race MRI changes were provisionally identified as cytokine family members: IL1RN, IL1*α* and IL8. Also tentatively identified were positive acute-phase proteins including C-reactive protein (CRP) and SAA which have been shown to increase during bouts of exercise, presumably to promote healing of damaged tissues [36].

As with any other analytical technique, SELDI-TOF has limitations [37].

 Not all proteins can be visualized equally well with SELDI-TOF; the range below 20 kDa is especially well resolved while sensitivity for higher molecular weight proteins is lower. Detection of high intensities of protein peaks of a specific molecular mass may not necessarily mean that high levels of the corresponding protein product will be present in body fluids due to the suppression of signals through higher-affinity binding, so that absolute quantization is not possible. However, as long as similar biological fluids are compared this is not an issue for biomarker discovery. Standardized sample processing is crucial to reproducible and robust SELDI-TOF analysis and we tried to minimize variation as described in Section 2. Analytical bias was minimized by running the samples in only two batches with several overlapping samples and analysis was performed on the overall dataset with a consistent baseline subtraction, normalization, alignment and noise reduction strategy. We are currently in the process of identifying the candidate proteins using a fractionation and nano LC/MS/MS strategy leading to verifying their significance with more quantitative ELISA assays. This study has for the first time identified potentially measurable biomarkers that could form the basis for a predictive test for significant cardiac damage during a marathon race.

At this stage, we cannot speculate further on the significance of these results and the mechanisms involved until positive identification of the proteins is made and the differential expression data has been verified. There could be many explanations for the diversity changes we observed (Table 1) that are involved in the mechanism of the response (acute and recovery) to exercise-induced cardiac stress. As examples, patterns 1 and 2 would seem to be involved in the acute response of proteins during and immediately after the race, pattern 3 may indicate an inflammatory response, and patterns 4 and 5 may indicate a recovery response. It is important to note that, although these patterns were the predominant change amongst the runners, there was individual variation amongst runners exemplified in Figure 2. The use of SELDI-TOF has uncovered not only potential proteins that may be involved in the acute and recovery phases of marathon running but has discovered proteins whose pre-race levels may predispose runners to higher risks of running-associated cardiac injury. This is the first step in the development of biomarker profiles that could be used on a more regular basis to indicate the individual risk of running a marathon.

## Figures and Tables

**Table 1:** Classification and prevalence of different patterns of protein change before, at the finish line, and after the race.

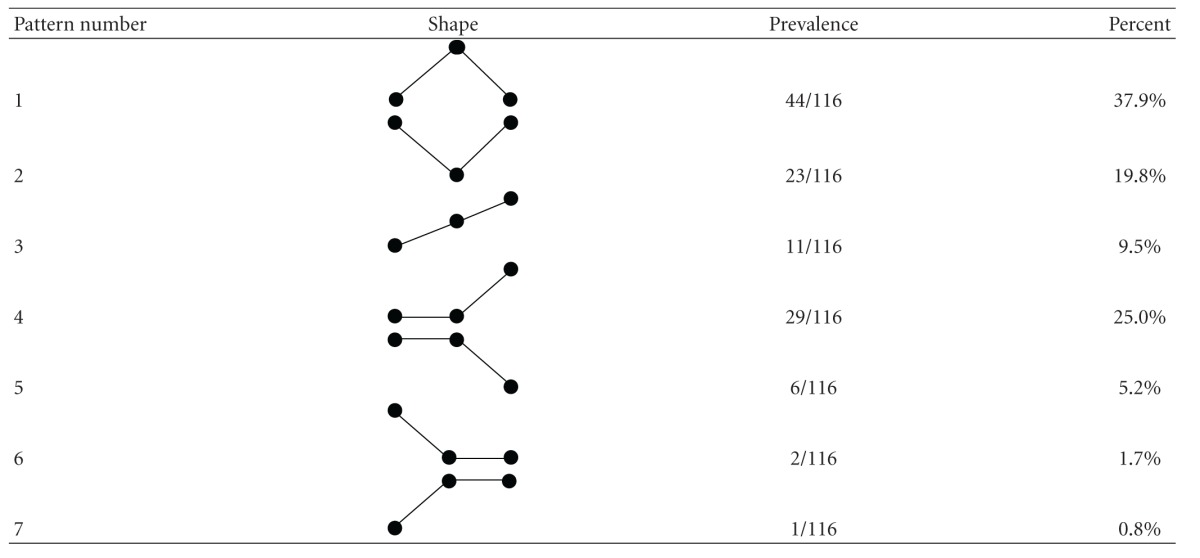

**Table 2:** Preliminary identification of candidate proteins significantly associated with RV EF changes pre-race, at the finish line and post-race.

* m/z* ratio	Pattern	Change	*P* value	Name	Protein ID
Pre-race protein analysis					
17772.3	2	1.79	0.0426	None	
17897.5	2	3.38	0.0074	IL1RN	Interleukin-1 receptor agonist protein
				Protein CX	Endothelial-overexpressed lipopolysaccharide-associated factor 1
18036.4	2	2.16	0.0168	IL1A	Interleukin-1 alpha (precursor)
				IL8	Interleukin-8 (precursor)
8922.3	2	0.70	0.0089	CCL8	C-C motif chemokines 8
16865.8	3	2.16	0.0230	none	

Finish line protein analysis					
10806.6	1	1.86	0.0004	S100A8	Calgranulin-A
10837.1	1	1.78	0.0047	S100A8	Calgranulin-A
13285.2	1	1.95	0.0015	IL13 SELH	Interleukin-13 (precursor) Selenoprotein H
13252.4	1	2.17	0.0015	None	
22283.7	1	0.82	0.0241	None	
22203.0	1	0.80	0.0155	CLCF1	Cardiotrophin-like cytokine factor 1
3375.45	1	0.73	0.0085	DEF3	Neutrophil defensin 3
10753.8	1	1.91	0.0053	CCKN	Cholecystokinin
16706.3	5	0.53	0.0003	CALM1 IL1F10	Calmodulin Interleukin-1 family member 10
12671.7	1	2.01	0.0055	SPEG	Striated muscle preferentially expressed protein kinase
10899.6	1	1.81	0.0101	None	
32038.5	1	0.78	0.0144	FCN1	Ficolin-1
116572	2	0.58	0.0149	CORIN	Atrial natriuretic peptide-converting enzyme
				LIPE	Hormone-sensitive lipase
10418.6	1	2.00	0.0006	CRP	C-reactive protein (precursor)
				S100A1	Protein S100-A1
				CASP1	Caspase-1 (precursor)
				SUMO4	Small ubiquitin-related modifier 4
185233	3	0.34	0.0423	None	
10970.5	1	1.73	0.0201	DEFA6	Defensin-6 (precursor)

Post-race protein analysis					
11654.2	4	1.38	0.0224	FKBP1B	Peptidyl-prolyl-cis-transisomerase FKBP1B (precursor)
				SAA1	Serum amyloid A protein (precursor)
				GPHA2	Glycoprotein hormone alpha-2 (precursor)
11603.7	4	1.35	0.0378	SAA1	Serum amyloid A protein (precursor)
11629.3	4	1.40	0.0224	SAA1	Serum amyloid A protein (precursor)
11699.7	4	1.39	0.0052	SAA1	Serum amyloid A protein (precursor))
11699.7	4	1.39	0.0052	KCNE3	Potassium voltage-gated channel subfamily E member 3
11885.4	4	1.59	0.0083	NPPB	Natriuretic peptide B (precursor
11320.9	4	1.59	0.0454	IL8	Interleukin-8 (precursor)
17772.3	2	0.67	0.0232	None	
17453.6	2	0.72	0.0126	IL18P	Interleukin-18-binding precursor (precursor)
17578.4	2	0.67	0.0092	TREM1	Triggering receptor expressed on myeloid cells 1
				IL26	Interleukin-26 (precursor)
17375.4	2	0.71	0.0194	IL1B	Interleukin-1-beta
				IL7	Interleukin-7 (precursor)
9709.5	2	1.32	0.0335	CHGA	Chromogranin A (precursor)
17381.1	2	0.70	0.0207	IL1B IL7	Interleukin-1 beta (precursor) Interleukin-7 (precursor)
10418.6	1	1.57	0.0426	CRP	C-reactive protein
				S100A1	Protein s100-A1active
				CASP1	Caspase-1 (precursor)
				SUMO4	Small ubiquitin-related modifier 4 (precursor)
11207.8	4	2.09	0.0452	SAA1	Serum amyloid A protein (precursor)
11052.8	3	1.45	0.0424	None	
